# Ultrasound Assessment of Internal Jugular Vein Distensibility as a Marker of Residual Congestion in Acute Decompensated Heart Failure

**DOI:** 10.3390/life16091570

**Published:** 2026-09-19

**Authors:** Andreea-Maria Grigore, Mihai Grigore, Andra-Valeria Hadarag, Camelia Nicolae, Ioana Verde, Ana-Maria Balahura, Adriana-Mihaela Ilieșiu

**Affiliations:** 1 Cardio-Thoracic Department, Carol Davila University of Medicine and Pharmacy, 021021 Bucharest, Romania; andreea-maria.banciu@drd.umfcd.ro (A.-M.G.); camelia.nicolae@umfcd.ro (C.N.); ana-maria.balahura@umfcd.ro (A.-M.B.); adriana.iliesiu@umfcd.ro (A.-M.I.); 2 Cardiology Department, Colentina Clinical Hospital, 020125 Bucharest, Romania; 3 Internal Medicine and Cardiology Department, “Prof. Th. Burghele” Clinical Hospital, 050653 Bucharest, Romania; hadaragandra@yahoo.com (A.-V.H.); ioana.verde@umfcd.ro (I.V.)

**Keywords:** acute decompensated heart failure, residual congestion, internal jugular vein, venous ultrasound, inferior vena cava, portal vein pulsatility, NT-proBNP

## Abstract

**Background**: Residual systemic congestion is common after treatment for acute decompensated heart failure (ADHF) and remains difficult to assess non-invasively. We investigated internal jugular vein distensibility (DIJV) as a dynamic marker of residual congestion and its association with established congestion parameters and clinical outcomes. **Methods**: In this prospective single-center study, 112 patients hospitalized with ADHF underwent clinical, laboratory, echocardiographic, and venous ultrasound assessment at admission and after decongestive treatment. DIJV was calculated as the ratio of maximal internal jugular vein diameter during Valsalva to resting diameter, with DIJV ≥ 4 indicating preserved distensibility. **Results**: At the final in-hospital assessment, 59 patients (52.7%) had persistent DIJV < 4. These patients had higher NT-proBNP, larger inferior vena cava diameter, greater portal vein pulsatility, higher pulmonary artery systolic pressure, and lower TAPSE/PASP compared with patients with preserved DIJV. They also had longer hospital stays and higher rates of ADHF rehospitalization and six-month mortality. Higher admission DIJV remained associated with lower odds of six-month ADHF rehospitalization after multivariable adjustment (OR 0.31 per 1-unit increase, 95% CI 0.15–0.59; *p* < 0.001). **Conclusions**: DIJV improves with decongestive treatment, but frequently remains impaired. Persistent impairment is associated with a broader residual-congestion profile and adverse clinical outcomes, supporting DIJV as a simple, dynamic adjunct to multiparametric congestion assessment.

## 1. Introduction

Congestion is a central feature of acute decompensated heart failure (ADHF), and incomplete decongestion at the end of hospitalization is associated with early rehospitalization and adverse prognosis [1]. Clinical signs of systemic venous congestion are often nonspecific and may become less apparent after treatment, even when elevated venous pressures persist. Jugular venous distension is particularly relevant because it reflects right-sided filling pressure, but bedside assessment may be challenging and subject to considerable interobserver variability. Venous ultrasonography provides a non-invasive and repeatable means of assessing systemic congestion and can complement clinical examination and conventional echocardiography. The internal jugular vein (IJV) is highly compliant at low venous pressure and normally expands substantially during the Valsalva maneuver; as venous pressure increases, baseline venous dimensions increase and the distensibility response becomes progressively blunted. Therefore, IJV distensibility may provide a simple dynamic marker of intravascular congestion.

The present study focused on the behavior of IJV distensibility during in-hospital decongestion, its relationship with established clinical, biochemical, and ultrasound congestion markers, and its association with subsequent clinical outcomes.

The objectives of this study were to assess changes in internal jugular vein distensibility (DIJV) from admission to the final in-hospital assessment following decongestive treatment in patients with ADHF; to evaluate the relationship between DIJV and established markers of congestion, including NT-proBNP, echocardiographic parameters, IVC diameter and collapsibility, and portal vein pulsatility; and to explore the association between DIJV and clinical outcomes, including ADHF rehospitalization and six-month mortality.

## 2. Materials and Methods

### 2.1. Study Design and Population

In this prospective, single-center observational study, patients hospitalized between 1 November 2022 and 1 January 2024 were screened for inclusion.

Patients were eligible if they were admitted with acute worsening of previously diagnosed chronic heart failure. The diagnosis of acute decompensated heart failure (ADHF) was established in accordance with the 2021 European Society of Cardiology Guidelines for the diagnosis and treatment of acute and chronic heart failure. An NT-proBNP concentration ≥ 300 pg/mL was required for inclusion [2]. No restriction was applied according to left ventricular ejection fraction.

Patients presenting with cardiogenic shock, septic shock, acute coronary syndrome-associated heart failure, or acute myocarditis were excluded. Additional exclusion criteria comprised heart failure related to primary severe valvular heart disease, severe chronic obstructive pulmonary disease or precapillary pulmonary hypertension, advanced liver disease (Child–Pugh class C), severe chronic kidney disease (glomerular filtration rate < 15 mL/min/1.73 m^2^) or dialysis, severe anemia (hemoglobin < 7 g/dL), and malignancy associated with a life expectancy of less than one year.

All enrolled patients were treated with intravenous loop diuretics during the first 48 h following admission. Clinical assessment, laboratory investigations, echocardiography, and venous ultrasonography were performed within the first 24 h of hospitalization. These evaluations were repeated following decongestive treatment, at the final available in-hospital assessment.

Written informed consent was obtained from all participants. The study was conducted in accordance with the Declaration of Helsinki and national recommendations for good medical practice. The study protocol was approved by the Ethics Committee of “Prof. Dr. Theodor Burghele” Clinical Hospital Bucharest, Romania (approval no. 9115/26 September 2022).

### 2.2. Clinical and Laboratory Assessment

All patients underwent a standard clinical evaluation at the time of hospitalization, including medical history, relevant comorbidities, current pharmacological treatment, presenting symptoms, and physical examination.

Laboratory assessment included NT-proBNP, hemoglobin, blood urea nitrogen (BUN), serum creatinine, sodium, and potassium. NT-proBNP was measured at baseline and reassessed following decongestive treatment. A decrease of >30% from the baseline value was considered a significant biochemical response [3].

Renal function was assessed using the estimated glomerular filtration rate (eGFR), calculated according to the 2021 CKD-EPI creatinine-based equation. Worsening renal function (WRF) was defined as an increase in serum creatinine > 0.3 mg/dL between the two study assessments [3].

The in-hospital furosemide dose was calculated as the total cumulative dose administered during hospitalization divided by the number of treatment days and is reported as mg/day.

### 2.3. Echocardiography and Venous Ultrasonography

Ultrasound examinations were performed at admission and repeated at the final in-hospital assessment. All examinations were conducted by two cardiologists, both certified in transthoracic echocardiography by the European Association of Cardiovascular Imaging (EACVI). The examiners were aware of the clinical context of the patient, and assessment was therefore not blinded. Venous measurements were, however, obtained and recorded before the corresponding NT-proBNP result for that time point was available, and all follow-up events occurred after the imaging assessments had been completed and recorded, so that outcome ascertainment was independent of the ultrasound findings. To assess inter-observer reproducibility, IJV resting and Valsalva diameters were independently remeasured by the second cardiologist, blinded to the original values, in a random subset of 20 patients. Agreement was quantified using the two-way random-effects, absolute-agreement intraclass correlation coefficient with 95% confidence intervals, and by Bland–Altman analysis reporting bias and 95% limits of agreement. Intra-observer reproducibility was assessed in the same subset after an interval of at least four weeks.

#### 2.3.1. Echocardiography and Inferior Vena Cava Assessment

Transthoracic echocardiography was performed using a GE Vivid E95 ultrasound platform (GE Vingmed Ultrasound AS, Horten, Norway) equipped with a 1.4–4.6 MHz transducer, with continuous ECG monitoring during image acquisition. The echocardiographic evaluation included left ventricular systolic function, assessment of left ventricular filling, right ventricular systolic function, inferior vena cava (IVC) dimensions and respiratory dynamics, and estimation of pulmonary artery systolic pressure (PASP).

For IVC evaluation, patients were examined in the supine position using a subcostal long-axis view. Measurements were obtained approximately 1–2 cm proximal to the IVC–right atrial junction. The maximum IVC diameter at end-expiration was recorded, together with the degree of inspiratory collapse elicited by a brief sniff maneuver. Respiratory collapsibility > 50% was considered preserved. An IVC diameter > 21 mm associated with <50% inspiratory collapse was considered indicative of elevated right atrial pressure [4].

#### 2.3.2. Internal Jugular Vein Ultrasonography

Ultrasonographic assessment of the internal jugular vein (IJV) was performed on the right side, with the patient positioned semi-supine at approximately 45°. The transducer was initially placed at the angle of the mandible and subsequently advanced caudally along the course of the sternocleidomastoid muscle toward the sternal angle. The right IJV was identified in transverse view adjacent to the common carotid artery, at approximately 5 cm below the mandibular angle [5]. Care was taken to avoid excessive transducer pressure that could alter the shape or dimensions of the vein.

IJV distensibility was assessed by comparing venous dimensions at rest with those obtained during the strain phase of the Valsalva maneuver [6]. At rest, the maximal transverse IJV diameter was measured at end-expiration. The measurement was then repeated at maximal venous distension during Valsalva. Diameter-based IJV distensibility (DIJV) was calculated as the ratio between the maximal transverse diameter during Valsalva and the resting end-expiratory diameter:

$$DIJV\;=\;\frac{IJV\;diameter\;during\;Valsalva\;}{resting\;end-expiratory\;IJV\;diameter}$$

According to previously reported thresholds, DIJV ≥ 4 was considered indicative of preserved distensibility, values from 2 to <4 of impaired distensibility, and DIJV < 2 of severely impaired distensibility. For the present study, patients were categorized according to the final in-hospital DIJV as having preserved (DIJV ≥ 4) or impaired (DIJV < 4) IJV distensibility [7]. As the ratio of two diameter measurements expressed in the same units, DIJV is dimensionless. Diameter-based DIJV was used as the main measure of IJV distensibility because it is simple to obtain and has shown good reproducibility [6,8].

#### 2.3.3. Portal Vein Ultrasonography

Portal venous flow was assessed using color and pulsed-wave Doppler, with the patient in the supine position and measurements obtained during end-expiratory apnea. The portal vein was visualized through a subxiphoid or transhepatic approach, and maximum (Vmax) and minimum (Vmin) flow velocities were recorded [9,10].

Portal vein pulsatility was quantified as:

$$PVPI\;=\;\frac{Vmax\;-\;Vmin\;}{\;Vmax}\times \;100$$

For the present analysis, a PVPI > 50% was used to identify a severely abnormal pulsatile portal venous flow pattern, in accordance with previously described thresholds [9,11]. The relative reduction in PVPI during hospitalization was calculated as $\frac{PVPI\;at\;admission\;-\;PVPI\;at\;the\;final\;in-hospital\;assessment}{PVPI\;at\;admission}$× 100. A reduction > 50% from the admission value was considered a marked decrease in portal vein pulsatility.

### 2.4. Clinical Outcomes

Clinical outcomes included in-hospital mortality, all-cause mortality within six months, and rehospitalization for acute decompensated heart failure (ADHF) during the six-month follow-up period. In-hospital mortality was defined as death from any cause occurring during the index hospitalization. Six-month mortality was defined as death from any cause occurring from the index hospitalization through six months of follow-up.

ADHF rehospitalization was defined as an unplanned hospital admission for worsening signs or symptoms of heart failure requiring inpatient treatment. Patients without available follow-up information were excluded.

### 2.5. Statistical Analysis

Descriptive and unadjusted statistical analyses were performed using IBM SPSS Statistics version 20 for Windows, whereas multivariable regression analyses were performed using R version 4.4.2. Continuous variables were summarized as mean ± standard deviation (SD) when approximately normally distributed and as median with interquartile range (IQR) otherwise. Normality was assessed using the Shapiro–Wilk test. Categorical variables were reported as counts and percentages. Comparisons between independent groups were performed using Student’s *t*-test for normally distributed continuous variables and the Mann–Whitney U test for non-normally distributed variables. Categorical variables were compared using the chi-square test or Fisher’s exact test, as appropriate. Changes between admission and the final in-hospital assessment were evaluated using the paired-samples *t*-test or Wilcoxon signed-rank test according to data distribution. Relationships between DIJV and continuous or ordinal variables, including NT-proBNP, echocardiographic parameters, IVC diameter, and PVPI were assessed using Spearman’s rank correlation coefficient. Patients were stratified according to DIJV measured at the final available in-hospital assessment (DIJV ≥ 4 vs. DIJV < 4). The association between admission DIJV and six-month ADHF rehospitalization was further evaluated using multivariable logistic regression. The primary adjusted model included admission DIJV, age, sex, and log2-transformed admission NT-proBNP. TAPSE, PASP, TAPSE/PASP, and IVC collapsibility were subsequently added separately to the primary model. TAPSE, PASP, and TAPSE/PASP were not entered simultaneously because TAPSE/PASP is mathematically derived from TAPSE and PASP. Results are reported as odds ratios (ORs) with 95% confidence intervals (CIs). Comparisons of DIJV values according to subsequent clinical outcomes were performed using the Mann–Whitney U test. Analyses were based on available data for each variable, without imputation of missing values; consequently, denominators may vary across individual analyses. All statistical tests were two-sided, and a *p*-value < 0.05 was considered statistically significant.

## 3. Results

**Figure 1:**
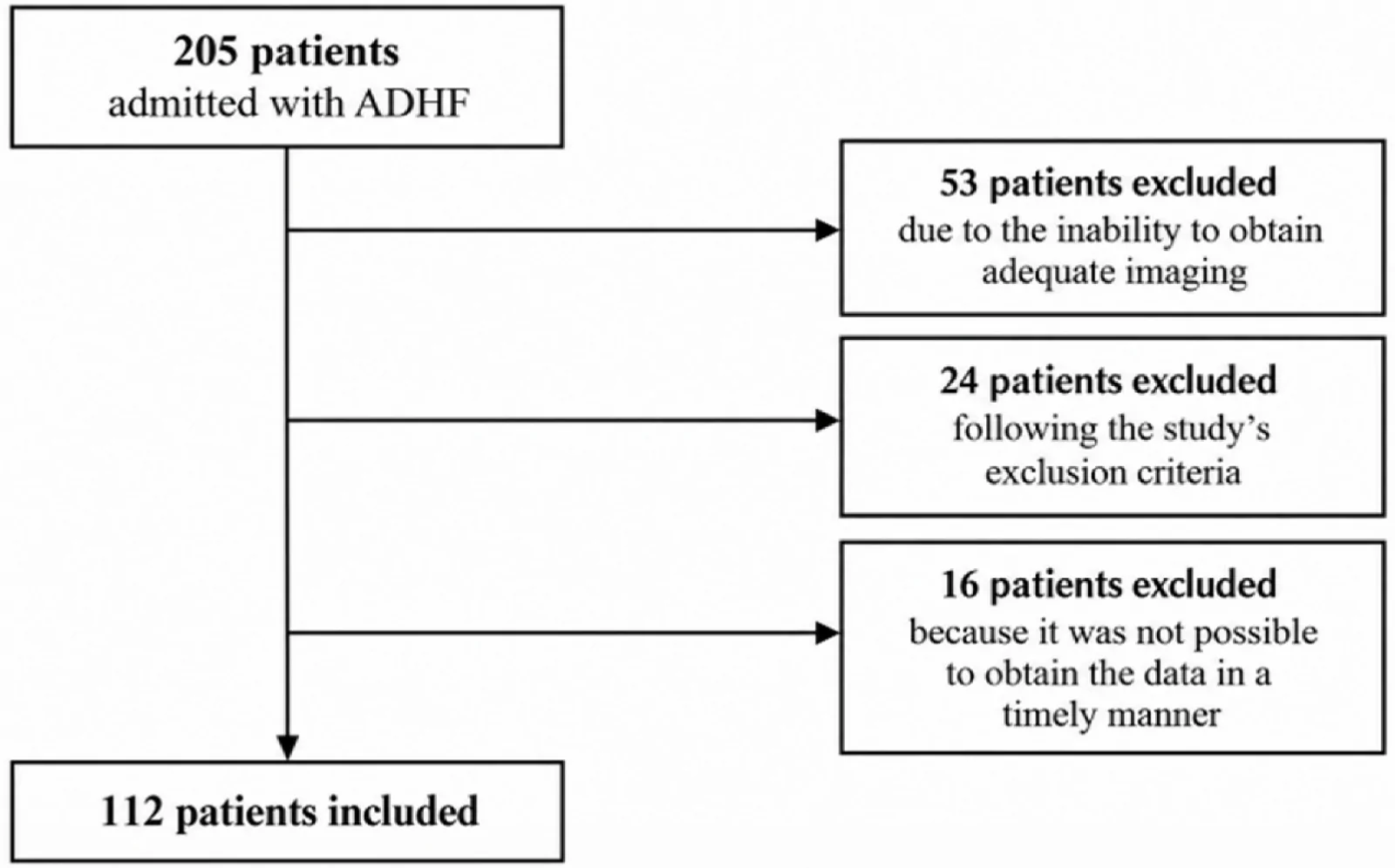
Patient selection flow diagram.

A total of 205 patients were hospitalized with acute decompensated heart failure (ADHF), of whom 112 were included in the final study cohort (Figure 1). Patients were excluded because of failure to meet the eligibility criteria, inadequate ultrasound imaging, or inability to complete the study assessments within the predefined timeframe. Imaging exclusions were driven by poor cardiac acoustic windows, not by the feasibility of jugular venous imaging.

### 3.1. Baseline Characteristics

Patients were stratified according to DIJV at the final in-hospital assessment: 53 patients had a DIJV ≥ 4 (Group 1), whereas 59 had a DIJV < 4 (Group 2). Baseline characteristics according to final DIJV category are presented in Table 1.

In the overall cohort, the median age was 74 years (IQR 67–82), 68 patients (60.7%) were male, median LVEF was 49% (IQR 34–59), and mean BMI was 29.4 ± 5.7 kg/m^2^. Age, sex, heart rate, systolic blood pressure, BMI, and LVEF did not differ significantly between groups, although patients with a final DIJV < 4 tended to be older than those with a DIJV ≥ 4 [74 (70–84) vs. 71 (64–79) years, *p* = 0.052]. Because reduced venous compliance is an expected consequence of vascular aging, the contribution of age to DIJV was examined further. Age was not associated with DIJV at admission (rho = −0.139, *p* = 0.146), but was weakly associated with DIJV at the final in-hospital assessment (rho = −0.279, *p* = 0.003), and was not associated with the magnitude of change in DIJV during hospitalization (rho = −0.104, *p* = 0.276). Older age was associated with a larger residual resting IJV diameter at the final assessment (rho = 0.252, *p* = 0.008) but not with the diameter achieved during Valsalva (rho = −0.014, *p* = 0.887). Hypertension (83.0%), chronic kidney disease (61.6%), atrial fibrillation (58.0%), anemia (55.4%), ischemic heart disease (39.3%), diabetes mellitus (36.6%), and chronic obstructive pulmonary disease (22.3%) were common, with no significant differences between the two groups. At admission, beta-blocker therapy was more frequent among patients with a final DIJV ≥ 4 than among those with a DIJV < 4 (94.3% vs. 76.3%, *p* = 0.008), whereas the use of ACE inhibitors, ARBs, ARNIs, MRAs, and SGLT2 inhibitors was similar between groups. Almost all patients were receiving loop diuretic therapy before hospitalization. During hospitalization, patients with a final DIJV < 4 received a higher median daily dose of furosemide than those with a DIJV ≥ 4 [160 (120–200) vs. 120 (80–160) mg/day, *p* < 0.001].

### 3.2. Changes in Clinical, Biochemical, Echocardiographic, and Venous Ultrasound Parameters During Hospitalization

Clinical, biochemical, echocardiographic, and venous ultrasound parameters at admission and at the final in-hospital assessment are presented in Table 2.

DIJV increased from 3.18 (IQR 2.57–3.82) at admission to 3.94 (IQR 3.42–4.40) at the final in-hospital assessment (*p* < 0.001). Resting IJV diameter decreased from 6.43 ± 1.27 to 4.50 ± 0.76 mm, whereas the Valsalva diameter decreased proportionally less (19.79 ± 2.74 to 17.04 ± 2.88 mm).

At admission, median NT-proBNP was 4136 pg/mL (IQR 1932–6954), median IVC diameter was 22.0 mm (IQR 19.0–24.0), median PVPI was 47.1% (IQR 36.1–59.0), and mean DIJV was 3.22 ± 0.80. Several differences were already present at admission between patient groups subsequently classified according to their final DIJV. Patients with a final DIJV < 4 had a higher PVPI (50.1% vs. 44.4%, *p* = 0.013). TAPSE/PASP was also lower in this group (0.32 vs. 0.36 mm/mmHg, *p* = 0.024). Admission NT-proBNP was numerically higher among patients with a final DIJV < 4, although the difference did not reach statistical significance (4688 vs. 3142 pg/mL, *p* = 0.076). Other clinical, biochemical, and echocardiographic parameters were similar between groups at admission (Table 2).

At the final in-hospital assessment, patients with DIJV < 4 had higher NT-proBNP (2090 vs. 1290 pg/mL, *p* < 0.001), and PASP (41.0 vs. 33.0 mmHg, *p* = 0.002), together with lower TAPSE (16 vs. 18 mm, *p* = 0.018) and TAPSE/PASP (0.35 vs. 0.45 mm/mmHg, *p* = 0.018). Venous ultrasound findings included a larger IVC diameter (21.0 vs. 16.0 mm, *p* < 0.001) and a higher PVPI (25.4% vs. 18.7%, *p* = 0.007). Body weight, systolic blood pressure, heart rate, hemoglobin, eGFR, and serum sodium did not differ significantly between groups (Table 2).

An NT-proBNP reduction > 30% occurred more frequently in patients with DIJV ≥ 4 than in those with DIJV < 4 (83.0% vs. 55.8%, *p* = 0.002). Similarly, a PVPI reduction >50% was more frequent in patients with DIJV ≥ 4 than in patients with DIJV < 4 (68.8% vs. 44.0%, *p* = 0.014), and preserved IVC collapsibility (>50%) at discharge was likewise more frequent in patients with DIJV ≥ 4 than in patients with DIJV < 4 (92.7% vs. 61.0%, *p* < 0.001).

Median length of stay was shorter in patients with DIJV ≥ 4 (8 [IQR 5–10] vs. 10 [IQR 8–12] days, *p* = 0.004), whereas worsening renal function did not differ significantly between groups (41.2% vs. 47.3%, *p* = 0.528) (Table 3).

### 3.3. DIJV and Other Congestion Markers

To further characterize the relationship between DIJV and congestion status, we examined its association with established biochemical, echocardiographic, and venous ultrasound markers of congestion. These analyses included NT-proBNP and its changes during hospitalization, echocardiographic parameters, inferior vena cava assessment, and portal vein pulsatility.

#### 3.3.1. DIJV and Changes in NT-proBNP During Hospitalization

**Figure 2:**
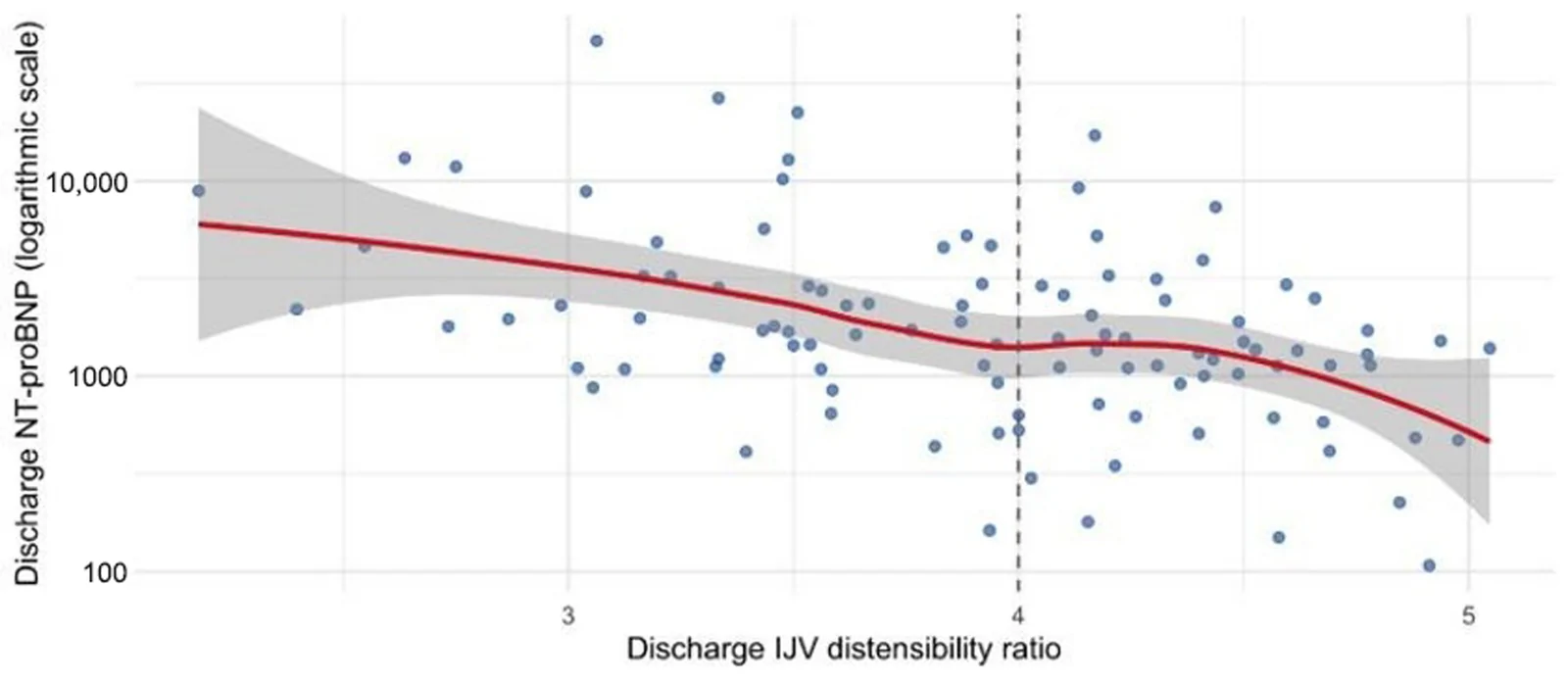
Correlation between DIJV and NT-proBNP at discharge. Each blue dot represents one individual patient; NT-proBNP is plotted on a logarithmic scale. The red line describes the average relationship between DIJV and NT-proBNP across the observed range of DIJV values, and the surrounding gray shaded area is the 95% confidence interval. The vertical dashed line marks the DIJV threshold of 4 used to define preserved distensibility.

At the final in-hospital assessment, DIJV was inversely correlated with contemporaneous NT-proBNP levels (r = −0.409, *p* < 0.001) (Figure 2). Patients with DIJV < 4 had significantly higher NT-proBNP levels than those with DIJV ≥ 4 (2090 [IQR 1204–4642] vs. 1290 [IQR 620–1960] pg/mL, *p* < 0.001) (Figure 2).

#### 3.3.2. DIJV and Echocardiographic Parameters

At discharge, DIJV was inversely correlated with PASP (r = −0.414, *p* < 0.001). Patients with DIJV < 4 had higher PASP (41.0 [IQR 34.0–48.0] vs. 33.0 [IQR 24.2–42.2] mmHg, *p* = 0.002). TAPSE was lower in patients with DIJV < 4 (16 ± 4 vs. 18 ± 3 mm, *p* = 0.018), as was the TAPSE/PASP ratio (0.35 [IQR 0.27–0.47] vs. 0.45 [IQR 0.38–0.57] mm/mmHg, *p* = 0.018) (Table 2).

#### 3.3.3. DIJV and Inferior Vena Cava

**Figure 3:**
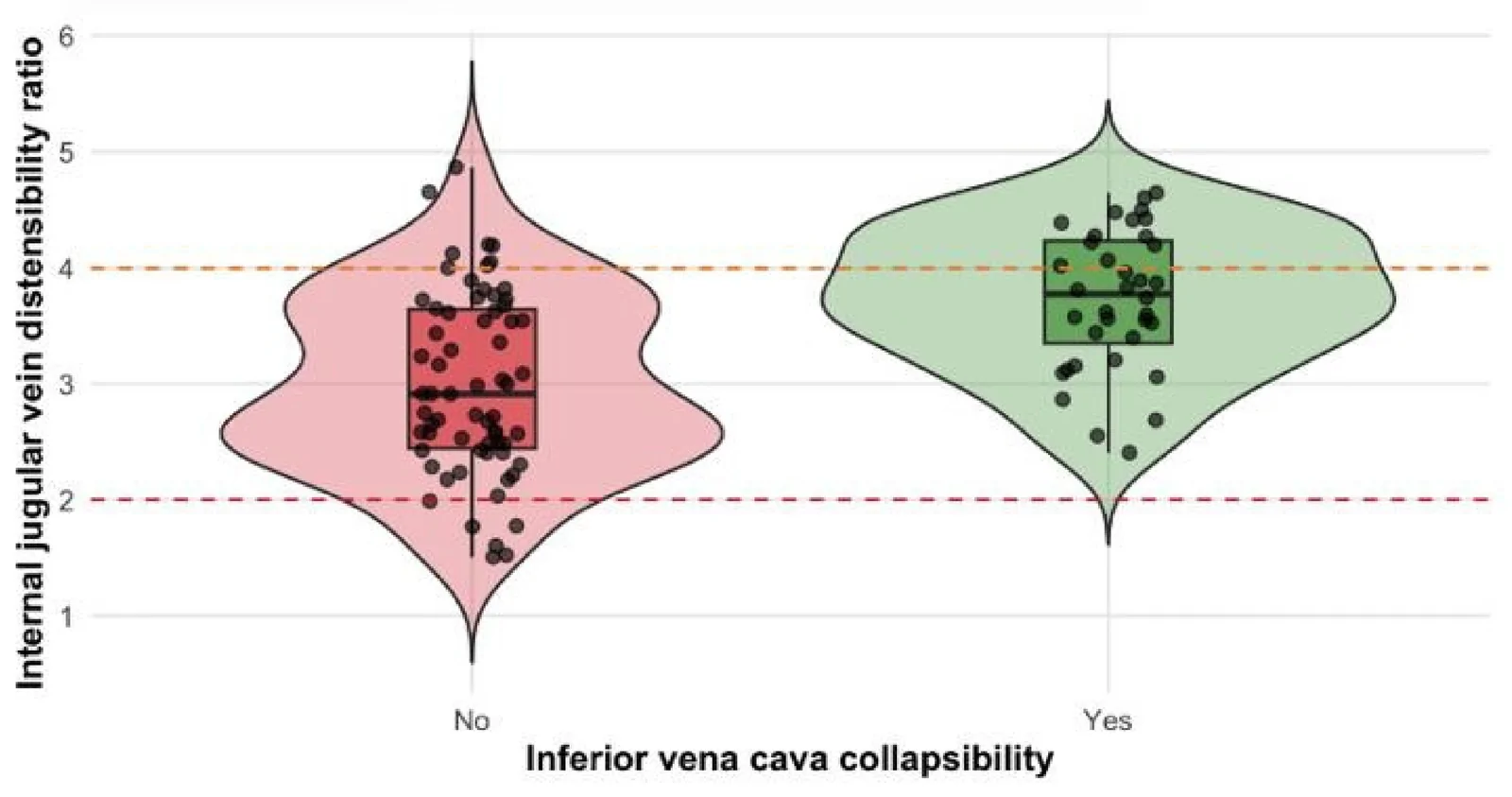
DIJV according to IVC collapsibility at admission. The red group on the left includes patients with reduced IVC collapsibility (IVC collapse ≤ 50%, “No”) and the green group on the right patients with preserved IVC collapsibility (IVC collapse > 50%, “Yes”). The violin plot shows how the patients were spread out: the width of the colored area at any given height is proportional to the number of patients with that DIJV value. The orange dashed line marks the DIJV threshold of 4 (normal distensibility) and the dark-red dashed line the threshold of 2 (severely impaired distensibility).

At admission, patients with preserved IVC collapsibility (>50%) had higher DIJV than those with reduced IVC collapsibility (3.77 [IQR 3.35–4.23] vs. 2.91 [IQR 2.46–3.64], *p* < 0.001) (Figure 3). At discharge, DIJV was inversely correlated with IVC diameter (r = −0.446, *p* < 0.001). Consistently, patients with DIJV < 4 had a larger IVC diameter than those with DIJV ≥ 4 (21.0 [IQR 16.0–24.0] vs. 16.0 [IQR 15.0–17.0] mm, *p* < 0.001) (Table 2). Preserved IVC collapsibility (>50%) at discharge was present in 60.98% of patients with DIJV < 4 compared with 92.68% of those with DIJV ≥ 4 (*p* < 0.001) (Table 3).

#### 3.3.4. DIJV and Portal Vein Pulsatility

At discharge, DIJV was inversely correlated with PVPI (r = −0.339, *p* < 0.001). Patients with DIJV < 4 had a higher PVPI than those with DIJV ≥ 4 (25.4% [IQR 14.1–46.6] vs. 18.7% [IQR 9.3–26.7], *p* = 0.007) (Table 2). A reduction in PVPI >50% during hospitalization was also less frequent among patients with DIJV < 4 than among those with DIJV ≥ 4 (44.00% vs. 68.75%, *p* = 0.014) (Table 3).

### 3.4. DIJV and Clinical Outcomes

During the index hospitalization and subsequent six-month follow-up, 16 patients (14.3%) died, including seven in-hospital deaths. Rehospitalization for ADHF occurred in 45 patients with available follow-up data.

#### 3.4.1. DIJV and Six-Month Rehospitalization

Rehospitalization for ADHF was more frequent among patients with DIJV < 4 at discharge than among those with DIJV ≥ 4 (61.54% vs. 24.53%, *p* < 0.001). DIJV measured at admission was also significantly lower in patients who were subsequently rehospitalized compared with those who were not (2.69 [IQR 2.41–3.36] vs. 3.64 [IQR 2.92–4.02], *p* < 0.001).

#### 3.4.2. DIJV and Six-Month Mortality

**Figure 4:**
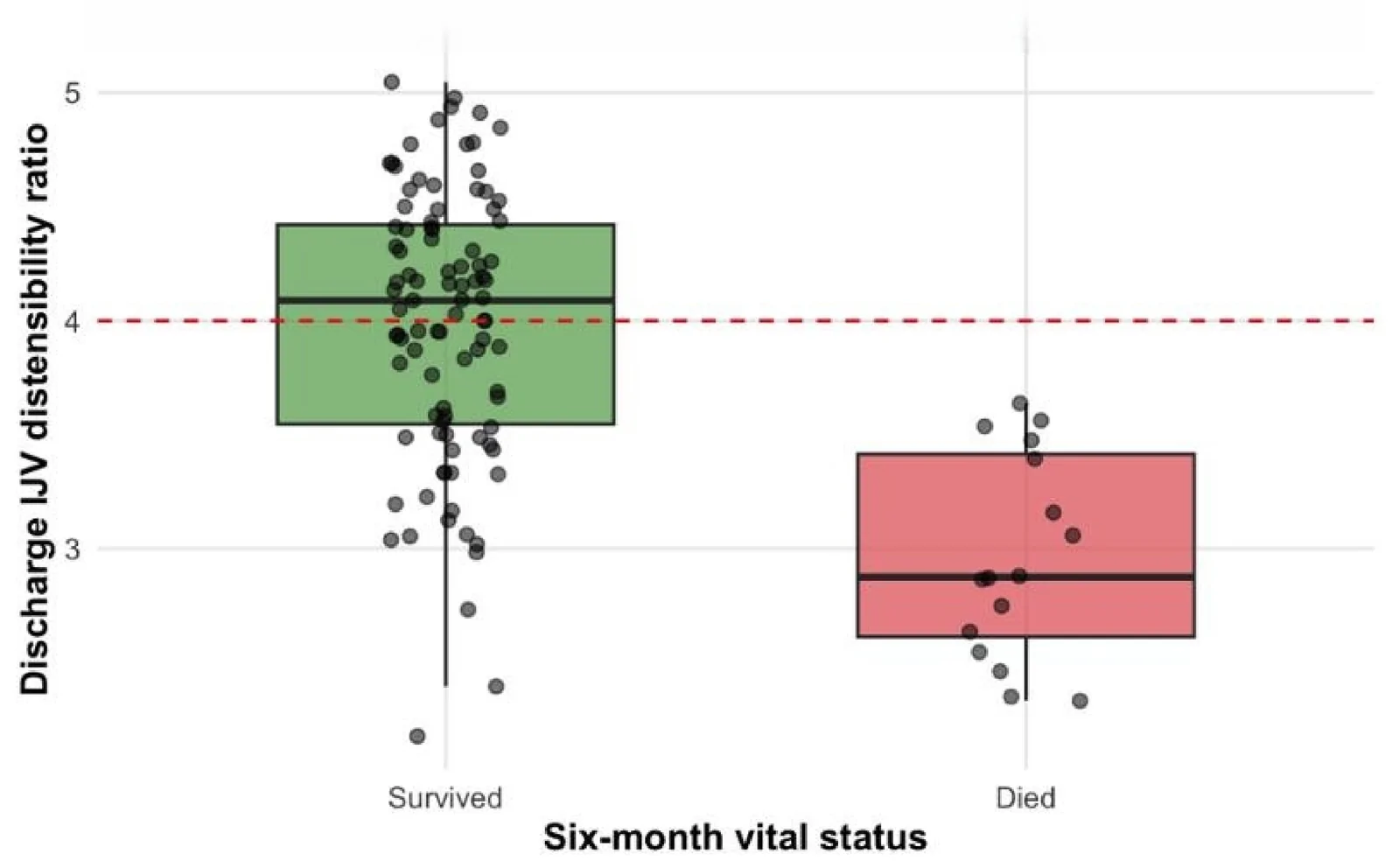
DIJV at discharge according to six-month vital status. The green box on the left represents patients who were alive at six months and the red box on the right patients who died within six months. Each black dot is one patient; the red dashed horizontal line marks the DIJV threshold of 4 used to define preserved distensibility.

Six-month mortality was higher among patients with DIJV < 4 at discharge than among those with DIJV ≥ 4 (27.1% vs. 0%, *p* < 0.001) (Figure 4).

#### 3.4.3. Multivariable Analysis

In multivariable logistic regression, higher admission DIJV remained associated with lower odds of six-month ADHF rehospitalization after adjustment for age, sex, and admission NT-proBNP (OR 0.31 per 1-unit increase, 95% CI 0.15–0.59; *p* < 0.001). The association remained significant when TAPSE, PASP, TAPSE/PASP, or IVC collapsibility were separately added to the primary adjusted model (Table 4).

ORs are reported per 1-unit increase in admission DIJV. The primary model included age, sex, and log2-transformed admission NT-proBNP; TAPSE, PASP, TAPSE/PASP, and IVC collapsibility were added separately. Complete-case analysis was used.

DIJV, internal jugular vein distensibility; OR, odds ratio; CI, confidence interval; TAPSE, tricuspid annular plane systolic excursion; PASP, pulmonary artery systolic pressure; IVC, inferior vena cava.

## 4. Discussion

The main findings of this study are that IJV distensibility is frequently impaired at admission in patients hospitalized with ADHF and increases during decongestive treatment, while remaining abnormal in a substantial proportion of patients at the final in-hospital assessment. Persistent impairment of DIJV was associated with a broader residual-congestion profile, characterized by higher NT-proBNP, larger IVC diameter, greater portal vein pulsatility, higher PASP, and less favorable right ventricular–pulmonary artery coupling. Patients with DIJV < 4 at the final in-hospital assessment also had longer hospital stays and higher rates of adverse clinical outcomes during follow-up. These findings support DIJV as a dynamic component of multiparametric congestion assessment rather than as a stand-alone diagnostic measure.

Systemic venous congestion is an important component of acute heart failure, and its persistence after decongestive treatment has been associated with an increased risk of subsequent adverse events [12]. Although jugular venous distension is traditionally assessed by physical examination, clinical estimation of jugular venous pressure is subject to considerable interobserver variability and may underestimate elevated filling pressures [13]. Ultrasound assessment of the IJV provides an objective approach to characterizing jugular venous dynamics and may therefore complement conventional clinical assessment.

The physiological behavior of DIJV reflects the pressure–volume characteristics of the venous system. At lower central venous pressure, the IJV is relatively small at rest and expands substantially during the Valsalva maneuver. As venous pressure increases, the vein becomes progressively distended at rest, reducing its capacity for further expansion during Valsalva [14]. In our cohort, decongestive treatment was accompanied by a marked reduction in resting IJV diameter, whereas the reduction in Valsalva diameter was less pronounced, resulting in an increase in DIJV at the final in-hospital assessment. This dynamic response provides a physiological rationale for serial assessment of DIJV during decongestive treatment.

DIJV was also associated with established markers of congestion. At admission, lower DIJV was associated with reduced IVC collapsibility and higher NT-proBNP. At the final in-hospital assessment, lower DIJV correlated with larger IVC diameter, higher PASP. Patients with DIJV < 4 also had higher NT-proBNP and a less favorable echocardiographic and venous ultrasound profile than those with DIJV ≥ 4. Previous studies in ambulatory and hospitalized patients with heart failure have similarly reported associations between abnormal jugular venous dynamics, natriuretic peptide concentrations, pulmonary pressures, and right-sided cardiac dysfunction [14,15]. Taken together, these findings suggest that DIJV reflects a component of systemic venous congestion that overlaps with, but is not interchangeable with, conventional biochemical, echocardiographic, and venous ultrasound markers.

An important observation was the persistence of impaired DIJV after decongestive treatment, with more than half of the cohort remaining below the predefined threshold of 4 at the final in-hospital assessment. This finding is relevant in the context of residual congestion, which remains common despite clinical improvement during hospitalization. However, because no invasive hemodynamic reference standard was available, the frequency of abnormal DIJV cannot be interpreted as evidence of greater sensitivity or diagnostic superiority over IVC or portal venous assessment. Rather, the persistence of abnormal DIJV, together with its associations with other markers of congestion, supports its potential complementary role within a multiparametric assessment [16].

The association between DIJV and clinical outcomes provides an additional observation of interest. Lower DIJV values were observed in patients who experienced ADHF rehospitalization or died during follow-up, and six-month mortality occurred exclusively among patients with DIJV < 4 at the final in-hospital assessment. Previous studies have also associated impaired jugular venous distensibility with adverse outcomes in both hospitalized and ambulatory heart failure populations [7,17]. Nevertheless, differences in clinical setting, congestion severity, ultrasound methodology, and follow-up duration may account for variability in the prognostic performance reported across studies. Importantly, the association between admission DIJV and ADHF rehospitalization persisted after adjustment for age, sex, and admission NT-proBNP, and remained significant when TAPSE, PASP, TAPSE/PASP, or IVC collapsibility were separately incorporated into the adjusted model. These findings suggest that the observed association is not explained solely by these measured markers of disease severity and congestion. However, the present analysis does not establish causality, superiority over established congestion measures, or incremental predictive value.

These findings further emphasize the multidimensional nature of congestion. IVC size and collapsibility, portal venous pulsatility, natriuretic peptides, echocardiographic indices, and jugular venous distensibility reflect related but distinct components of cardiac loading conditions and systemic venous congestion. Accordingly, discordance between individual markers is not unexpected and may favor a multiparametric rather than single-marker approach [18,19,20,21,22,23]. Serial DIJV assessment is non-invasive and readily repeatable at the bedside, but its incremental clinical value beyond established congestion markers remains to be established. Further prospective multicenter studies should determine whether DIJV improves assessment of residual congestion and risk stratification after decongestive treatment.

## 5. Limitations

This study should be interpreted in light of several limitations. The single-center design and relatively small cohort limit the generalizability of the findings. Invasive hemodynamic assessment was not available to validate DIJV against a reference standard, while Valsalva-dependent measurements may be affected by patient cooperation and technical variability. Additionally, ultrasound assessment was not blinded to the patient’s clinical status. These findings therefore require confirmation in larger prospective multicenter cohorts.

## 6. Conclusions

DIJV improved during decongestive treatment in patients hospitalized with ADHF but remained impaired in a substantial proportion at the final in-hospital assessment. Lower DIJV was associated with biochemical, echocardiographic, and venous ultrasound markers of residual congestion, as well as with adverse clinical outcomes during follow-up. These findings support DIJV as a simple, dynamic, and complementary marker within a multiparametric assessment of congestion. Further prospective studies are needed to establish its incremental clinical and prognostic value.

## Figures and Tables

**Table 1:** Clinical characteristics and treatment according to DIJV category.

Baseline	Total (112 Patients)	Group 1: DIJV ≥ 4 (*n* = 53)	Group 2: DIJV < 4 (*n* = 59)	*p* Value
Age, years, median (IQR)	74 (67–82)	71 (64–79)	74 (70–84)	0.052
Male sex, *n* (%)	68 (60.71%)	31 (58.49%)	37 (62.71%)	0.648
HR, beats/min, median (IQR)	76 (70–100)	75 (69–110)	77 (70–100)	0.768
SBP, mmHg, median (IQR)	130 (114–148)	130 (120–149)	130 (110–146)	0.090
BMI, kg/m^2^, mean (±SD)	29.4 (±5.7)	29.3 (±5.7)	29.5 (±5.6)	0.851
IHD (%)	44 (39.29%)	20 (37.74%)	24 (40.68%)	0.750
Hypertension, *n* (%)	93 (83.04%)	46 (86.79%)	47 (79.66%)	0.315
Diabetes mellitus, *n* (%)	41 (36.61%)	17 (32.08%)	24 (40.68%)	0.345
Atrial fibrillation, *n* (%)	65 (58.04%)	28 (52.83%)	37 (62.71%)	0.290
Chronic kidney disease, *n* (%)	69 (61.61%)	29 (54.72%)	40 (67.80%)	0.155
Anemia, *n* (%)	62 (55.36%)	25 (47.17%)	37 (62.71%)	0.099
COPD, *n* (%)	25 (22.32%)	11 (20.75%)	14 (23.73%)	0.706
LVEF (%), median (IQR)	49 (34–59)	50 (35–60)	48 (33–56)	0.355
ACEi admission (%)	37 (33.04%)	17 (32.08%)	20 (33.90%)	0.838
ARB admission (%)	18 (16.07%)	10 (18.87%)	8 (13.56%)	0.445
ARNI admission (%)	20 (17.86%)	11 (20.75%)	9 (15.25%)	0.448
Beta-blocker admission (%)	95 (84.82%)	50 (94.34%)	45 (76.27%)	0.008
MRA admission (%)	78 (69.64%)	39 (73.58%)	39 (66.10%)	0.390
SGLT2i admission (%)	39 (34.82%)	20 (37.74%)	19 (32.20%)	0.539
Loop diuretic before admission (%)	108/111 (97.3%)	52/53 (98.1%)	56/58 (96.6%)	1.000
Thiazide before admission (%)	2 (1.79%)	0 (0.00%)	2 (3.39%)	0.497
Furosemide in hospital (mg/day), median (IQR)	120 (80–200)	120 (80–160)	160 (120–200)	<0.001

HR—heart rate; SBP—systolic blood pressure; BMI—body mass index; IHD—ischemic heart disease; COPD—chronic obstructive pulmonary disease; LVEF—left ventricular ejection fraction; ACEi—angiotensin-converting enzyme inhibitor; ARB—angiotensin II receptor blocker; ARNI—angiotensin receptor–neprilysin inhibitor; MRA—mineralocorticoid receptor antagonist; SGLT2i—sodium–glucose cotransporter 2 inhibitor; IQR—interquartile range; SD—standard deviation.

**Table 2:** Clinical, biochemical, echocardiographic, and venous ultrasound findings at admission and final in-hospital assessment.

Parameters	Total (112)	Group 1: DIJV ≥ 4 (n = 53)	Group 2: DIJV < 4 (n = 59)	*p* Value	Total (112)	Group 1 (53)	Group 2 (59)	*p* Value
Time	Admission				Discharge			
Weight (kg)	83.6 (±17.9)	84.7 (±18.3)	82.6 (±17.7)	0.550	82.0 (±17.0)	83.2 (±17.2)	80.9 (±16.9)	0.483
SBP (mmHg)	130 (114–148)	130 (120–149)	130 (110–146)	0.090	115 (110–120)	120 (110–120)	115 (105–120)	0.551
HR (beats/min)	76 (70–100)	75 (69–110)	77 (70–100)	0.768	70 (65–77)	69 (65–70)	70 (65–78)	0.084
Hemoglobin (g/dL)	12.2 (±2.7)	12.2 (±2.8)	12.2 (±2.5)	0.930	12.7 (±2.5)	13.0 (±2.5)	12.5 (±2.6)	0.259
eGFR (mL/min/1.73 m^2^)	55.6 (±21.1)	57.7 (±20.4)	53.7 (±21.8)	0.322	58.7 (±19.1)	57.8 (±17.6)	59.5 (±20.7)	0.665
Na (mmol/L)	139.0 (±3.7)	139.4 (±3.5)	138.7 (±3.8)	0.329	138.0 (135.0–140.0)	138.0 (135.0–139.5)	138.0 (134.0–140.2)	0.932
NT-proBNP (pg/mL)	4136 (1932–6954)	3142 (1340–5428)	4688 (2506–11,022)	0.076	1564 (1025–2902)	1290 (620–1960)	2090 (1204–4642)	<0.001
TAPSE (mm)	16 (±4)	17 (±3)	16 (±4)	0.115	17 (±4)	18 (±3)	16 (±4)	0.018
PASP (mmHg)	50.9 (±14.2)	48.3 (±13.5)	53.1 (±14.6)	0.079	37.9 (28.0–45.0)	33.0 (24.2–42.2)	41.0 (34.0–48.0)	0.002
TAPSE/PASP (mm/mmHg)	0.34 (0.25–0.40)	0.36 (0.29–0.42)	0.32 (0.23–0.38)	0.024	0.40 (0.34–0.53)	0.45 (0.38–0.57)	0.35 (0.27–0.47)	0.018
IVC diameter (mm)	22.0 (19.0–24.0)	22.0 (19.0–23.0)	22.0 (19.2–25.0)	0.579	17.0 (15.0–22.0)	16.0 (15.0–17.0)	21.0 (16.0–24.0)	<0.001
PVPI (%)	47.1 (36.1–59.0)	44.4 (33.3–55.6)	50.1 (40.1–59.8)	0.013	21.4 (10.9–35.0)	18.7 (9.3–26.7)	25.4 (14.1–46.6)	0.007

DIJV, internal jugular vein distensibility; SBP, systolic blood pressure; HR, heart rate; eGFR, estimated glomerular filtration rate; Na, serum sodium; NT-proBNP, N-terminal pro-B-type natriuretic peptide; TAPSE, tricuspid annular plane systolic excursion; PASP, pulmonary artery systolic pressure; IVC, inferior vena cava; PVPI, portal vein pulsatility index.

**Table 3:** Decongestion response according to DIJV category at discharge.

Outcome	Total	DIJV ≥ 4	DIJV < 4	*p* Value
Length of stay, days, median (IQR)	9 (7–12)	8 (5–10)	10 (8–12)	0.004
Worsening renal function, *n*/*N* (%)	47/106 (44.34%)	21/51 (41.18%)	26/55 (47.27%)	0.528
NT-proBNP decrease > 30%, *n*/*N* (%)	73/105 (69.52%)	44/53 (83.02%)	29/52 (55.77%)	0.002
PVPI decrease > 50%, *n*/*N* (%)	55/98 (56.12%)	33/48 (68.75%)	22/50 (44.00%)	0.014
Preserved IVC collapsibility (>50%) at final in-hospital assessment, *n*/*N* (%)	63/82 (76.83%)	38/41 (92.68%)	25/41 (60.98%)	<0.001
In-hospital death, *n* (%)	7 (6.25%)	0 (0.00%)	7 (11.86%)	0.014
All-cause mortality within 6 months, *n* (%)	16 (14.29%)	0 (0.00%)	16 (27.12%)	<0.001
ADHF rehospitalization during follow-up, *n*/*N* (%)	45/105 (42.86%)	13/53 (24.53%)	32/52 (61.54%)	<0.001

DIJV, internal jugular vein distensibility; NT-proBNP, N-terminal pro-B-type natriuretic peptide; PVPI, portal vein pulsatility index; IVC, inferior vena cava; IQR, interquartile range.

**Table 4:** Multivariable association between admission DIJV and six-month ADHF rehospitalization.

Model	N/Events	DIJV OR (95% CI)	*p* Value
Primary adjusted model	106/45	0.31 (0.15–0.59)	<0.001
Primary model + TAPSE	104/45	0.30 (0.15–0.59)	<0.001
Primary model + PASP	104/45	0.30 (0.14–0.58)	<0.001
Primary model + TAPSE/PASP	104/45	0.31 (0.15–0.61)	0.001
Primary model + IVC collapsibility	97/42	0.33 (0.15–0.66)	0.003

## Data Availability

Data used in this study may be provided by the authors upon reasonable request.

## Abbreviations

The following abbreviations are used in this manuscript:

ACEi	Angiotensin-Converting Enzyme Inhibitor
ADHF	Acute Decompensated Heart Failure
AF	Atrial Fibrillation
ARB	Angiotensin II Receptor Blocker
ARNI	Angiotensin Receptor–Neprilysin Inhibitor
BMI	Body Mass Index
BUN	Blood Urea Nitrogen
CKD	Chronic Kidney Disease
COPD	Chronic Obstructive Pulmonary Disease
DIJV	Internal Jugular Vein Distensibility Ratio
eGFR	Estimated Glomerular Filtration Rate
HF	Heart Failure
HR	Heart Rate
HTN	Hypertension
IHD	Ischemic Heart Disease
IJV	Internal Jugular Vein
IQR	Interquartile Range
IVC	Inferior Vena Cava
IVCc	Inferior Vena Cava Collapsibility
JVD	Jugular Venous Distension
LVEF	Left Ventricular Ejection Fraction
MRA	Mineralocorticoid Receptor Antagonist
NT-proBNP	N-Terminal Pro-B-Type Natriuretic Peptide
PASP	Pulmonary Artery Systolic Pressure
PV	Portal Vein
PVPI	Portal Vein Pulsatility Index
SBP	Systolic Blood Pressure
SD	Standard Deviation
SGLT2i	Sodium–Glucose Cotransporter 2 Inhibitor
TAPSE	Tricuspid Annular Plane Systolic Excursion
VM	Valsalva Maneuver
WRF	Worsening Renal Function
